# Comorbidity Profile and Predictors of Obstructive Sleep Apnea Severity and Mortality in Non-Obese Obstructive Sleep Apnea Patients

**DOI:** 10.3390/medicina59050873

**Published:** 2023-05-01

**Authors:** Dragana Milicic Ivanovski, Branka Milicic Stanic, Ivan Kopitovic

**Affiliations:** 1 Faculty of Medicine, University of Novi Sad, Hajduk Veljkova 3, 21000 Novi Sad, Serbia; 2 Department of Medicine, Georgetown University Medical Center, 4000 Reservoir Road, NW, Washington, DC 20057, USA; 3 Center for Pathophysiology of Breathing and Respiratory Sleep Disorders, The Institute for Pulmonary Diseases of Vojvodina, Put dr Goldmana 4, 21204 Sremska Kamenica, Serbia

**Keywords:** obstructive sleep apnea, comorbidity profile, mortality, disease severity, non-obese patients, cardiovascular risk

## Abstract

*Backgrounds and Objectives:* Obstructive sleep apnea (OSA) is associated with increased morbidity and mortality. OSA is an independent risk factor for many different conditions, especially cardiovascular diseases. The purpose of this study was to ascertain the comorbidity profile of non-obese patients with newly diagnosed OSA and evaluate the risk for cardiovascular disease and mortality. The present study also aimed to establish predictors for OSA severity. *Materials and Methods:* This study included 138 newly diagnosed patients who underwent polysomnographic analysis. The 10-year risk for cardiovascular disease was assessed using a newly validated prediction model: Systematic Coronary Risk Evaluation (SCORE-2). In addition, the Charlson Comorbidity Index (CCI) was assessed as a widely-used example of a mortality comorbidity index. *Results:* The study population included 138 patients: 86 males and 52 females. Patients were stratified, according to AHI (apnea/hypopnea index), into four groups: 33 patients had mild OSA (5 ≤ AHI < 15), 33 patients had moderate OSA (15 ≤ AHI < 30), 31 patients had severe OSA (AHI ≥ 30), and 41 individuals had AHI < 5, which were a part of the control group. SCORE-2 increased in line with OSA severity and was higher in OSA groups compared to the control group (H = 29.913; DF = 3; *p* < 0.001). Charlson Index was significantly higher in OSA patients compared to controls (*p* = 0.001), with a higher prevalence of total comorbidities in the OSA group of patients. Furthermore, CCI 10-year survival score was significantly lower in the OSA group, suggesting a shorter survival of those patients with a more severe form of OSA. We also examined the prediction model for OSA severity. *Conclusions:* Determining the comorbidity profile and estimation of the 10-year risk score of OSA patients could be used to classify these patients into various mortality risk categories and, according to that, provide them with adequate treatment.

## 1. Introduction

Obstructive sleep apnea (OSA) is the most important breathing disorder during sleep. 

OSA is an often inadequately diagnosed condition characterized by repeated episodes of apnea or hypopnea caused by partial or complete obstruction of the upper airways during sleep, a decrease in blood oxygen saturation, and daytime sleepiness [1,2,3]. Sleep apnea syndrome occurs in 2–26% of the general population, depending on gender, age, and body mass index. In middle-aged men, it is present in 4% of cases, and in women, in 2% of cases. The prevalence in asymptomatic people can be up to 24% and up to 60% in the elderly and obese [2,4]. One of the most important mechanisms of OSA is the presence of intermittent hypoxemia, followed by reoxygenation, which leads to oxidative stress. As a result, systemic inflammation develops through molecules that cause damage to blood vessel endothelial cells, which contributes to atherosclerosis and the occurrence of cardiovascular diseases in obstructive sleep apnea [1,4,5]. 

Constant sympathetic activation, reduction of baroreceptor sensitivity, and release of vasoactive mediators that change the function of vascular endothelium are believed to trigger hypertension in obstructive sleep apnea [6]. Age, a high Body Mass Index (BMI), and male sex are all risk factors for OSA [7,8]. 

Metabolic, cardiovascular, renal, pulmonary, and neuropsychiatric comorbidities are frequently linked to obstructive sleep apnea (OSA). Although there is growing evidence that some of these comorbidities may induce OSA, there is still strong evidence that OSA is an independent risk factor for many of these conditions [9]. Additionally, it has been shown that severe OSA raises mortality from all causes [10]. As a result, there is mounting proof that OSA and comorbidities are linked bidirectionally, particularly in the cases of heart failure, metabolic syndrome, and stroke [9]. Patients with OSA are at high risk for several cardiovascular diseases: hypertension, ischemic heart disease, atrial fibrillation, cerebrovascular disease, and heart failure. Thirty-five–70% of patients with OSA have hypertension, while approximately 30% of patients with hypertension have OSA [3]. It is crucial to remember that people with OSA not only have a greater likelihood of developing cardiovascular comorbidities but also suffer worse outcomes from cardiovascular diseases [11]. 

Pathophysiological mechanisms in obstructive sleep apnea potentiate the emergence or worsening of an already present metabolic imbalance, primarily carbohydrate and fat metabolism, with the development of metabolic syndrome Z, which represents the sum of metabolic syndrome X associated with sleep apnea [1,12]. 

Oxidative stress in OSA affects the development of metabolic disorders such as diabetes, dyslipidemia, insulin, and leptin resistance [4,13,14]. 

Obstructive sleep apnea associated with chronic obstructive pulmonary disease (COPD) is an overlap syndrome that occurs in 10–20% of patients with OSA [15]. The presence of OSA in COPD contributes to the worsening of respiratory insufficiency during the night, which worsens the clinical course of COPD [16]. 

Repeated hypoxemia, sleep fragmentation, and reduction of slow-wave sleep in patients with obstructive sleep apnea contribute to hypoxic-ischemic damage of the brain and damage of cerebral circulation, which can subsequently lead to stroke, dementia, and mental disorders. [17,18]. 

Comorbidities in OSA patients are crucial since they significantly affect their use of healthcare resources and have a big impact on mortality [19,20]. OSA and comorbidities together may increase the cardiometabolic risk, worsening morbidity and mortality [9]. Untreated severe OSA patients experience a greater incidence of fatal and non-fatal cardiovascular events compared with primary snorers or OSA patients who received CPAP treatment [21].

The aim of this study was to determine the comorbidity profile of non-obese patients with newly diagnosed obstructive sleep apnea and evaluate the risk for cardiovascular disease, which can help stratify them according to the risk for fatal and non-fatal cardiovascular disease and the risk for mortality. The present study also aimed to establish predictors for OSA severity. 

## 2. Material and Methods

This was an observational, cross-sectional study that included a total of 138 patients who were referred to the Center for Pathophysiology of Breathing and Respiratory Sleep Disorders at the Institute for Pulmonary Diseases of Vojvodina for a polysomnographic examination. The study was conducted between 2018 and 2022 and included patients over 18 years of age of both sexes.

The Ethics Committee of the University of Novi Sad, Faculty of Medicine, approved the study. Every procedure conducted during this investigation complied with the ethical guidelines established by the institution’s Ethics Committee and with the Declaration of Helsinki. Informed written consent was obtained from all participants included in the study. Exclusion criteria were patients with central sleep apnea, sleeping disorders like parasomnia and insomnia and/or neurological diseases, psychiatric disease, malignancies, patients with BMI > 30, pregnant women, and patients with previous diagnoses or treatment of OSA.

The diagnosis of obstructive sleep apnea was established by polysomnographic analysis. The study group included 97 patients with polysomnographicaly confirmed obstructive sleep apnea (AHI > 5) and 41 people in whom the presence of obstructive sleep apnea was ruled out by polysomnography (AHI < 5), which were a part of the control group.

The following data were collected: demographic (gender, age), anthropometric characteristics (BMI, neck circumference, waist circumference, Mallampati configuration of the oral cavity and pharynx), and presence of the following comorbidities: hypertension, coronary disease, history of myocardial infarction, heart failure, hyperlipidemia, diabetes, hypothyroidism, asthma, chronic obstructive pulmonary disease, gastroesophageal reflux disease, allergic rhinitis, deviation of the nasal septum, occurrence of snoring, and data regarding sleep habits and smoking.

Using the Epworth Sleepiness Scale (ESS), daytime sleepiness was evaluated, which was completed by all subjects. It is a self-administered questionnaire evaluating the possibility of falling asleep in a variety of situations (maximum score is 24; score > 10 indicative of excessive daytime sleepiness). All patients also completed the Stop Bang questionnaire, which consists of 8 questions and starts with the acronym “STOP BANG” (Snoring, Tired, Observed—refers to the cessation of breathing, blood Pressure—arterial pressure, BMI—body mass index, Age, Neck circumference, Gender). Scoring is based on yes/no responses, with “yes” marked as 1 and “no” as 0. The score range is 0–8, and the cut-off value is ≥3. 

**Figure 1:**
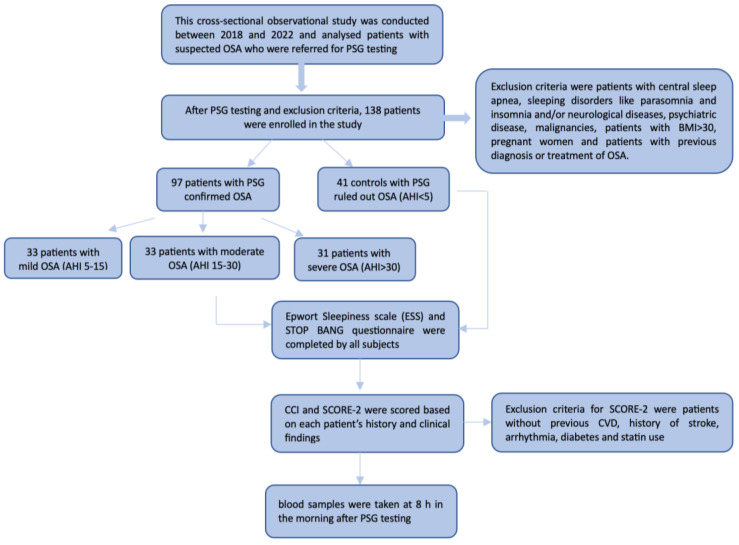
Flow diagram of the protocol study.

In addition, the interviewer scored CCI and SCORE-2 based on each patient’s history and clinical findings. The exclusion criteria for SCORE-2 were patients without previous cardiovascular disease (CVD), history of stroke, arrhythmias, diabetes, and statin use.

(Charlson Comorbidity Index) CCI is a well-validated and widely-used example of a mortality comorbidity index [22]. Each of the 19 conditions or comorbidities is given a weighted score of one through six, summed to a final Charlson Index score (range 0–37). This scoring method enables quick evaluation of the severity of a patient’s chronic disease and predicts their risk of mortality [23]. Flow diagram of the protocol study is presented on Figure 1.

### 2.1. Cardiovascular Risk Assessment 

The 10-year risk for cardiovascular disease was assessed using a newly validated prediction model: Systematic Coronary Risk Evaluation (SCORE-2). It is an updated prediction model using expected incidence and risk factor distribution according to country-specific CVD mortality. According to that, Serbia is in a very high-risk region for CVD events. The SCORE-2 estimates the 10-year risk of fatal and non-fatal cardiovascular diseases in individuals in Europe without previous CVD or diabetes using an algorithm that considers parameters such as age, gender, total cholesterol, high-density lipoprotein cholesterol, systolic blood pressure, and smoking status. When those variables are combined, SCORE-2 values indicate a 10-year risk for cardiovascular mortality that ranges from less than 1% to more than 15%, enhancing the identification of individuals at higher risk for developing CVD across Europe [24].

### 2.2. Polysomnography Assessment

All patients underwent all-night polysomnography (PSG) testing, during which the following parameters were recorded: electroencephalography, electrooculography, submental electromyography, lower leg electromyography, electrocardiography, leg movement, chest and abdominal movement, nasal airflow, pulse oximetry, body position, and snoring intensity. The polysomnographic recordings were manually scored according to the American Association of Sleep Medicine guidelines for the scoring of sleep and associated events. According to the guidelines, apnea was defined as a reduction in airflow of more than 90% for at least 10 s. Hypopnea was defined as a recognizable reduction of the airflow by more than 30% for at least 10 s, with a reduction in oxygen saturation of at least ≥ 3% from the baseline or arousal registered by the electroencephalography (EEG) [25]. A minimum of 4 h of total sleep was required to consider a study acceptable. AHI was calculated as the number of apnea/hypopnea events per hour of sleep time. Average, minimum, and maximum oxygen saturation and oxygen desaturation index during the sleep test were registered. The oxygen desaturation index (ODI) is the index of nocturnal hypoxemia and was defined as the value of oxygen desaturation per hour of sleep. Mean heart rate, minimum, and maximum heart rate were registered during PSG testing. 

Patients were categorized into four severity groups based on AHI: no OSA (reference, AHI < 5), mild OSA (5 ≤ AHI < 15), moderate OSA (15 ≤ AHI < 30), and severe OSA (AHI ≥ 30) [26]. 

### 2.3. Laboratory Analysis

Blood samples were taken at 8 h in the morning, after PSG assessment and 12 h of fasting. Blood gas analyses, fasting glucose, insulin, and lipid profile, including total cholesterol, low-density lipoprotein (LDL), high-density lipoprotein (HDL), non-HDL, triglycerides, VLDL, and atherosclerosis index, were measured. Furthermore, insulin resistance was measured by the homeostatic model assessment index of insulin resistance (HOMA-IR). HOMA-IR is calculated as the product of the fasting serum insulin concentration (mU/L) and fasting plasma glucose concentration (mmol/L) divided by 22.5 [27]. Three blood pressure measurements were taken 5–10 min apart using a standard, calibrated blood pressure sphygmomanometer while the participant was relaxed and in the sitting position. The average of the final two measures was used in the analyses. 

### 2.4. Statistical Analysis 

Arithmetic mean with standard deviation or median with a range that includes the 25th–75th percentile was used to describe continuous numerical variables. Attributive features are described using frequency distribution and percentages, i.e., absolute and relative numbers. Assessment of the normality of the distribution of continuous variables was done using the Kolmogorov-Smirnov and Shapiro-Wilk tests. To test the statistical significance of categorical variables, the chi-square test of independence or Fisher’s exact probability test was used. Also, in the chi-square test of independence for 2 × 2 tables, Yates’s correction was performed. To compare continuous variables with a normal distribution, Student’s *t*-test for independent samples was used, or one-factor analysis of variance (ANOVA) if it was a comparison of more than two groups. Post-hoc testing after analysis of variance was performed and included either Taki’s test or Bonferroni’s correction. Where the criterion of normality of distribution was not met, for the analysis of continuous variables, the Mann-Whitney test was used for comparing two groups and the Kruskal-Wallis test for testing more than two groups. After the Kruskal-Wallis test, the Mann-Whitney method was used for post hoc analysis. Multivariate analysis of variance (MANOVA) was used to compare groups with more than one characteristic. To investigate the relationship between variables, correlation was used, and multinomial logistic regression analysis was used as a technique for assessing the influence of a set of predictors on the dependent variable. For correlation testing, Pearson’s or Spearman’s correlation tests were used. All independent variables whose statistical significance was *p* < 0.05 were analyzed within multinomial logistic regression for the prediction model of the severity of sleep apnea as an outcome of interest. A univariate analysis was performed to check the statistical significance. The results of multinomial logistic regression are represented by a beta coefficient (Beta), standard error (Std. Error), and *p*-value. SPSS, version 26 (IBM SPSS, Armonk, New York, NY, USA) and Microsoft Excel 2019 (Microsoft, Redmond, WA, USA) programs were used for statistical data processing. The results are presented tabularly and graphically, and those values that reached the *p* < 0.05 level were considered statistically significant.

## 3. Results

The study population included 138 patients: 86 males and 52 females. Patients were stratified, according to AHI, into four groups: the control group of 41 patients had apnea-hypopnea index AHI < 5 (20 males and 21 females); 33 patients (17 males and 16 females) had mild OSA (AHI 5–14.9 events/hour); 33 patients (22 males and 11 females) had moderate OSA (AHI 15–29.9 events/h); and 31 patients (27 males and 4 females) had severe OSA (AHI > 30 events/h). The mean age of the control group was significantly lower than any subgroup of OSA patients (37.88 ± 14.31 for controls versus 53.39 ± 15.01 for mild OSA, versus 54.82 ± 11.95 for moderate OSA, and 53.97 ± 11.52 for severe OSA; F = 14.39; DF = 3; *p* < 0.001). In relation to BMI, a statistically significant difference was observed between the examined groups (F = 14.608; DF = 3; *p* < 0.001). Patients with severe OSA had higher BMI compared to the control group (28.10 ± 1.99 versus 24.25 ± 2.98, *p* < 0.001). A post hoc test determined the difference between the group without OSA and all other OSA groups, as well as between the mild OSA group and the severe OSA group. Forty patients were active smokers (29%). Anthropometric characteristics of all participants are presented in Table 1, while sleep characteristics and comparisons between groups are presented in Table 2.

In relation to diastolic blood pressure, statistically significant higher mean values were in the moderate and severe OSA groups, compared to controls (F = 3.461; DF = 3; *p* < 0.018). In regard to sleep characteristics, statistically significant differences were found for slow wave sleep, AHI in REM (rapid eye movement stage of the sleep) and NREM phase (non-rapid eye movement stage of the sleep), arousals index, minimum, and average oxyhemoglobin saturation, oxygen desaturation index, as well for Epworth Sleepiness scale and Stop Bang questionnaire, among the observed groups as shown in Table 2. 

Laboratory parameters are presented in Table 3. Statistically significant differences were found between the analyzed groups. In relation to HDL, significantly lower HDL values were observed in the severe OSA group compared to the mild OSA and control group (F = 5.745; DF = 3; *p* = 0.001). In relation to triglycerides, statistically significant higher values were noticed in severe and moderate OSA groups compared to the controls (H = 35.759; DF = 3; *p* < 0.001). In relation to VLDL, statistically significant differences were observed between the control and the moderate OSA groups (F = 4.466; DF = 3; *p* = 0.005). In regard to the value of glucose, insulin, and HOMA index, statistically significant differences were observed between the examined groups, controls in comparison to the moderate or severe OSA groups, as well as between mild and severe OSA groups (*p* < 0.001 for glucose; versus *p* = 0.004 for insulin; versus *p* = 0.002 for HOMA index). 

The representation of comorbidities by groups of participants is presented in Table 4. Regarding comorbidities, hyperlipidemia was the most prevalent disease, present in 63% of the study population, followed by hypertension (44.20% of the study population). 26.8% of the study population had deviated septum, and 24.6% had allergic rhinitis. 18.8% had GERD, asthma was present in 15.2% of patients, and 7.2% had diabetes mellitus. Hypothyroidism and COPD groups had the same value of 4.3%, and 2.9% of patients had a history of previous myocardial infarction, while heart failure and coronary disease were present in 2.2%. In relation to hypertension, hyperlipidemia, and diabetes mellitus, which were more prevalent in OSA patients, statistically significant differences were noticed between the control group compared to moderate and severe OSA groups, respectively (*p* < 0.001; *p* < 0.001; *p* = 0.015).

SCORE-2 increased in line with OSA severity, and statistically significant higher values were observed in the OSA group compared to the controls (H = 29.913; DF = 3; *p* < 0.001) (Table 5). 

**Figure 2:**
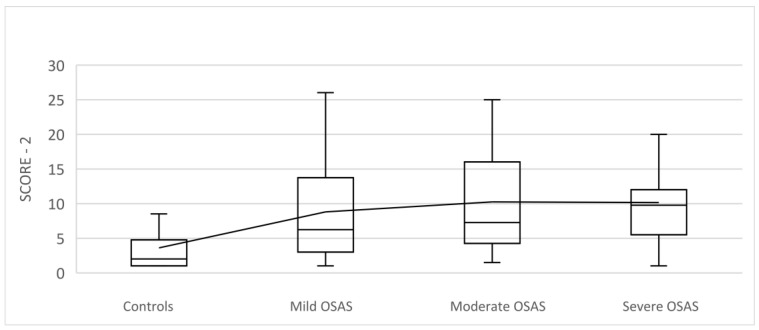
The bar graph with a comparison of SCORE-2 between controls and OSAS patients with mild (AHI 5–15), moderate (AHI 15–30), and severe OSA (AHI > 30). SCORE-2 increased in line with OSA severity, and statistically significant higher values were observed in the OSAS group of the patients compared to the controls (H = 29.913; DF = 3; *p* < 0.001). SCORE-2—Systematic Coronary Risk Evaluation.

A comparison of SCORE-2 between controls and OSA patients is presented in Figure 2. 

In addition, the Charlson Index was significantly higher in OSA patients compared to controls (*p* = 0.001). The OSA group was characterized by a higher prevalence of total comorbidities. As well, for CCI 10-year survival score, a statistically significant difference was observed between the examined groups (H = 16.489; DF = 3; *p* < 0.001). Post hoc test determined the difference between the controls and the OSA group (Table 6).

**Figure 3:**
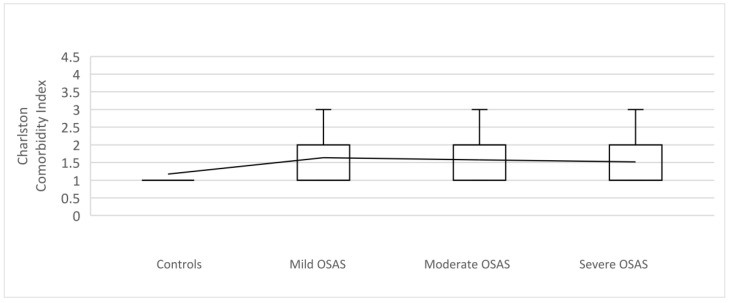
The bar graph with a comparison of the Charlson Comorbidity Index between controls and the OSAS group of the patients with mild (AHI 5–15), moderate (AHI 15–30), and severe OSA (AHI > 30). The Charlson Comorbidity Index was significantly higher in the OSAS group of the patients compared to controls (*p* = 0.001).

A comparison of the Charlson Comorbidity Index between controls and OSAS patients is presented in Figure 3.

Results with important correlations between SCORE-2, and Charlson Comorbidity score with anthropometric data and sleep characteristics, are presented in Table 7. 

The multivariate linear regression model was used for the prediction of obstructive sleep apnea severity (Table 8). In the complete model, all parameters that were initially statistically significant and described in the previously mentioned tables were taken into account. All significant parameters were tested for multicollinearity, and in the end, a total of 14 parameters entered the model: gender, age, ESS, Stop Bang, Mallampati, BMI, hypertension, snoring, pO_2_, pH value, HDL, triglycerides, HOMA index, and SCORE-2 index (note: linear interpolation was performed for SCORE-2 index to compensate for missing data). The whole model with all predictors was statistically significant, Chi-square (48, n = 138) = 175.652, *p* < 0.001, which shows that the model differentiates the respondents’ group affiliation (Cox and Snell R^2^ = 0.720; Nagelkerke R^2^ = 0.769; McFadden R^2^ = 0.461). In the complete model for the prediction of severe OSA, three parameters proved to be significant: gender, Mallampati score, and snoring. The odds ratio for gender (Exp(B) = 0.022) indicates that being female reduces the chance of a patient being in the severe sleep apnea group by a factor of 0.022. The odds ratio for the Mallampati score (Exp(B) = 5.297) indicates that a one-unit increase in the score raises the chance of a patient being in the severe sleep apnea group by a factor of 5.297. The odds ratio for snoring (Exp(B) = 61.042) indicates that the presence of snoring increases the chance of a patient being in the severe OSA group by a factor of 61.042. All data are presented in Table 8. 

## 4. Discussion

In this study with newly diagnosed OSA patients, we observed strong correlations, which are important as predictive factors for OSA severity. These results are in accordance with previous studies and can provide the discovery of important factors that lead to increased cardiovascular risk and mortality in OSA patients. 

Due to the existence of strong confounders, including obesity and hypertension, the importance of OSA as an independent risk factor has long remained debatable. Over the previous ten years, there has been an increase in publications on the topic of comorbidities in OSA, which indicates a growing interest in the contribution of comorbidities to OSA [28]. Obstructive sleep apnea is a condition that is commonly associated with an increased cardiovascular risk. Regardless of other significant cardiovascular risk factors, OSA is a well-known main risk factor for cardiovascular disability in terms of morbidity and mortality. The defining feature of OSA is intermittent hypoxia, which leads to oxidative stress and consequently generates systemic inflammation, increased lipid production, sympathetic hyperactivity, and endothelial dysfunction. All of these factors contribute to cardiometabolic comorbidities [10,29]. In a recent study, hypoxia, besides other polysomnography measures, was identified as a predictor of increased risk for cardiovascular disorders and mortality [30]. There are several possible mechanisms that could influence how OSA raises the risk of CVD [31,32]. Normal sleep is accompanied by a decrease in sympathetic nerve activity, metabolic rate, heart rate, and blood pressure and an increase in cardiac vagal tone during the non-rapid eye movement sleep stage, and an increase in sympathetic activity during rapid-eye movement sleep [32,33]. By disrupting this overall circulatory event during sleep, OSA may also affect the cascade of acute hemodynamic, metabolic, inflammatory, pharmacological, and autonomic reactions to sleep, which may contribute to worsening cardiovascular illnesses in people with OSA [34]. 

Clinical cohort studies and epidemiological data from the general population show that up to 50% of patients with arterial hypertension, refractory arrhythmias, stroke, coronary heart disease, and cardiac failure have OSA. Cardiometabolic abnormalities are detected in up to 50% of patients with OSAS as well [33,35,36]. Moreover, the presence of OSA and hypertension will have a more harmful impact on the cardiovascular system and may mutually worsen or trigger a variety of metabolic abnormalities [3,37]. Regarding our study, we observed a significantly higher number of patients with hypertension, hyperlipidemia, and diabetes mellitus among OSA patients in comparison to the control group of participants without OSA. We determined that the Epworth sleep scale, STOP bang questionnaire, Mallampati, and sleep parameters like ODI and arousals index were significantly higher in the OSA group, and slow-wave sleep was significantly lower in the OSA group compared to controls. In a variety of observational studies, measures of nocturnal hypoxemia, such as the proportion of the time spent sleeping with oxygen saturation below 90%, have been shown to more accurately predict the CVD risk and mortality than the AHI index [38,39]. Patients with OSA exhibit a lower percentage of slow-wave sleep than patients without OSA, even after adjusting for covariates in disease diagnostics [40]. In a study that analyzed the relationship between snoring and EEG signals by polysomnography on individuals with primary snoring and patients with OSAS, it was shown that snoring sounds affect deep restorative sleep (N3 delta wave). Those individuals who only have snoring and do not have OSAS may have difficulties with concentrating, forgetfulness and attention deficit, often with the absence of daytime sleepiness [41]. It is also reported impaired memory, intelligence, and selective attention in patients who have more than 3% of oxygen desaturation in the REM phase of sleep [42]. Ren et al. explored the correlation between slow-wave sleep and the prevalence of hypertension in OSA patients in a dose-dependent way [43]. In a cross-sectional investigation of primary snorers and patients with OSA, Zhang et al. discovered a connection between a decrease in slow-wave sleep and a risk for hypertension. After adjusting possible confounding variables, patients with lower slow-wave sleep and OSA had a significant increase in the incidence of hypertension [44]. As a result of our study, there is a direct correlation between OSA severity and increased CVD risk. OSA severity may have an impact on cardiovascular outcomes due to the cumulative effects of repeated ischemic reperfusion impairment brought on by obstructive respiratory episodes [45,46]. According to the performed study, there is a direct correlation between the number of cardiovascular risk factors and the AHI index. Significantly higher AHI values were seen in subgroups with a higher number of cardiovascular risk factors, as well as a higher incidence and severity of obstructive sleep apnea. Among the examined cardiovascular risk factors in the aforementioned study, only obesity, hypertension, and smoking were considered independent predictors of higher AHI values [47].

Stratification by sex showed that severe OSA is significantly associated with incident CVD in men but not in women. In this study, 42% of patients had hypertension, and 62% had hyperlipidemia, mainly males. In the study by Dudenbostel et al. was reported that obstructive sleep apnea affected 90% of male patients and 77% of female patients with resistant hypertension and that nocturnal hypertension is significantly more common in patients with OSA and is linked to an increased risk of heart failure, stroke, myocardial infarction, and total cardiovascular disease [48]. 

The Renin Aldosterone system (RAS) is activated in OSA patients due to intermittent or cyclical hypoxia [49]. According to a recent meta-analysis that investigated the link between OSA and RAAS activation in patients with and without hypertension separately, patients with hypertension and OSA had significantly higher aldosterone levels compared to those with hypertension but without OSA [50]. Also, it was discovered that individuals with OSA had lower serum 25-hydroxyvitamin D levels and higher PTH levels compared to healthy controls [51]. Since research by Stanic Milicic et al. indicates an independent relationship between the production of aldosterone and parathyroid hormone (PTH) in hypertensive patients [52], it is suggested that the determination of aldosterone and PTH could be introduced in all OSA patients with hypertension. Determining the wider metabolic profile would provide a comprehensive insight into increased cardiovascular risk in OSA patients and the timely introduction of adequate therapy.

In the study by Basoglu et al., lipid profiles were evaluated in patients with OSA after eliminating confounding factors such are obesity and diabetes mellitus. They found out that levels of total cholesterol, non-HDL, LDL cholesterol, and triglycerides were significantly higher in OSA patients compared to those without OSA and that impaired lipid profile was associated with OSA severity. We determined impaired lipid profile in non-obese patients with moderate and severe OSA compared to non-OSA individuals in terms of decreased HDL cholesterol and elevated levels of VLDL and triglycerides, while there was no significant difference in total cholesterol and LDL levels between compared groups [53]. For that reason, our results support the assumption that HDL cholesterol, VLDL, and triglyceride levels might be a better predictor for increased cardiometabolic risk in non-obese OSA patients as a part of atherogenic dyslipidemia.

Besides impaired lipid profile in OSA patients, it is proved that lipid peroxidation and protein oxidation in OSA significantly correlated with the severity of the disease. The lipid role seems to be prominent, considering that oxidative stress is involved in endothelial dysfunction and atherosclerotic development [21].

It is acknowledged that patients with OSA have a higher prevalence of diabetes than people without OSA [54]. On the other hand, people with severe OSA (e.g., AHI > 30) without initial diabetes mellitus (DM) are thought to have a higher risk of acquiring DM in the future, based on clinical and population-based observational cohort studies [55,56]. The main reason for inadequate plasma glucose and insulin balance in OSA patients is increased sympathetic activity [33]. In our study, we proved a higher prevalence of DM among OSA patients, with significantly higher values of glucose, insulin levels, and HOMA index. Insulin resistance has been identified as a crucial characteristic in the pathogenesis of OSA and hypertension, and it is essential for the onset and progression of both conditions. Yang et al. found that there is a strong association between insulin resistance and the risk for cardiovascular diseases in patients with OSA and hypertension and that the metabolic score for the insulin resistance index is important as an indicator of the occurrence of CVD and its subtypes. For that reason, it is very important to implement the detection of insulin resistance in the management of patients with OSA and hypertension as a preventive measure for CVD progression [57]. It is established that insulin resistance pathophysiologically changes lipid metabolism through oxidative stress, causing hyperlipidemia, hypertension, and other CV diseases [58,59]. 

Prevalence of diseases promoted by atherosclerosis (such as aneurysms, ischemic heart disease, and stroke) are more common in OSA patients. Consistent long-term clinical and epidemiological investigations have shown a higher risk of cardiovascular morbidity and mortality among individuals with untreated OSA. Additionally, epidemiological studies have shown that people with OSA who receive the appropriate CPAP treatment experience a decrease in cardiovascular risk [60]. 

In our study, the model for prediction of OSA severity had three variables entering the final model, namely: gender, Mallampati configuration, and occurrence of snoring, which have good predictive power for belonging to a severe sleep apnea group, with the important note that our study did not include obese patients. In research, Yan et al. established a similar risk prediction model for moderate-severe sleep apnea, which includes six variables entering the final prediction model, including BMI, hypertension, morning dry mouth, suffocating awake at night, witnessed apnea, and ESS total score. Their model has good consistency in predicting the occurrence of moderate to severe OSA [61]. Ye et al. used a machine learning diagnostic model based on the XGBoost algorithm for accurate prediction of children with different OSA severities, using heart rate and blood oxygen data as the major features. When compared to PSG, this diagnostic modality reduces the number of signals and uses a simpler diagnostic process which may be helpful for those with suspected OSA but does not have the opportunity of obtaining a diagnostic PSG [62]. 

In recent research, Maniaci et al. applied artificial intelligence with a clinical-based algorithm to predict the severity of OSA using clinical parameters, questionnaires, anatomical scores, and comorbidities. Their research demonstrated that dyslipidemia is the main predictor of OSA severity among included clinical variables [63]. 

OSA has consistently been linked in epidemiological research to decreased survival. According to a meta-analysis of 16 trials and 24,308 people, severe OSA (AHI > 30) is linked to higher all-cause and cardiovascular mortality [64]. A systematic review has proven the assumption that AHI is a predictor of all-cause mortality, and the authors found out that patients with severe OSA (AHI > 30) die at about twice the rate of controls. All analyzed studies that reported a higher risk of death in patients with moderate to severe OSA in their multivariate models included age and BMI, and most of them included smoking, sex, race, hypertension or blood pressure, and diabetes [65].

In our study, the Charlson Index was significantly higher in OSA patients compared to controls (*p* = 0.001), with a higher prevalence of total comorbidities in the OSA group of patients. Furthermore, CCI 10-year survival score was significantly lower in the OSA group, suggesting a shorter survival of those patients with a more severe form of OSA. Comparable to our results, Ruel et al. demonstrated an independent association between the existence of OSA and multimorbidity in a representative sample of men, which was dominant in men with moderate to severe OSA and three or more comorbidities [66]. Chiang et al. investigated comorbidities in patients with OSA and developed the CoSA (Comorbidities in Sleep Apnea) index score, which can help in the evaluation of mortality risk in OSA. The calculation for the CoSA index was similar to the Charlson Index, and comparing OSA patients with and without comorbidities, those with comorbidities had a greater likelihood of death (HR:11.01, 95% CI 4.00–30.33, *p* < 0.001). After multivariable adjustment, the chance of death was increased by age and 10 comorbid conditions, although the limitation of the study was that they did not include AHI value and information like BMI, smoking status, and severity of the diseases, which can confound the study [67]. 

A study that included 393 patients stratified according to the AHI index proved that the SCORE index rises with OSA severity. A positive correlation between the AHI and the SCORE was revealed, which was similar to our results [68]. 

In relation to all findings mentioned above, OSA is a complex chronic illness with several co-morbidities and pathogenic variables. Patients who exhibit different phenotypes may vary not just in their clinical manifestations but also in the pathogenic causes, underlying genetics, and outcomes of their diseases. This implies that the best mode of therapy may vary depending on the patient subgroup [69].

Our study has several advantages and limitations. It represents a strong correlation between established parameters that are responsible for increased CV risk and OSA severity. Innovations in the current study are using a novel SCORE-2 cardiovascular risk assessment in order to find a correlation between OSA severity and cardiovascular risk, as well as find predictors for OSA severity and mortality. According to the data in this research, we can stratify newly diagnosed OSA patients into various risk categories for developing cardiovascular disease and complications. The limitations of this study are that the comorbidities in the Charlson Index were collected on the self-reported forms and that details were confirmed by medical records. It is crucial to mention the fact that a single-night PSG was obtained and that it can influence the results due to altered sleep quality in hospital conditions. A small sample size in this study could be mentioned as one of the limitations; therefore, further research with a larger sample is necessary in order to increase the accuracy of provided correlations. There is also a need for future research, and a follow-up study would be beneficial in order to investigate the long-term influence of risk factors on cardiovascular morbidity, mortality, and outcomes in OSA patients. 

## 5. Conclusions

In summary, in a sample of individuals with newly diagnosed OSA and after excluding obesity as a most confounding factor, we have established that a 10-year risk for cardiovascular morbidity rises in concordance with the severity of OSA, as assessed by AHI. In order to identify and treat risk factors for cardiovascular disease and prevent future cardiovascular events in patients with OSA, sleep physicians should keep this data in mind. This study represents strong evidence of an association between established parameters and OSA severity, determining potential factors that are responsible for increased cardiovascular risk. Comorbidities in OSA patients have been linked to a greater risk of mortality. The mapping of the obstructive sleep apnea syndrome’s heterogeneity could contribute to the early detection of populations at risk. Determining the comorbidity profile of OSA patients could be used to classify these patients into various mortality risk categories and, according to that, provide them with adequate treatment. Last but not least, finding predictors of comorbidities and phenotypes in OSA suggests the necessity to include these elements when evaluating obstructive sleep apnea syndrome treatment strategy.

## Figures and Tables

**Table 1:** Comparison of anthropometric characteristics between controls and OSAS patients.

Variables	Controls (n = 41)	Mild OSAS (n = 33)	Moderate OSAS (n = 33)	Severe OSAS (n = 31)	Total of the Study Population (n = 138)	*p*-Value
Gender n (%)						**0.004**
Males	20 (48.8)	17 (51.5)	22 (66.7)	27 (87.1)	86 (62.3) *	
Females	21 (51.2)	16 (48.5)	11 (33.3)	4 (12.9)	52 (37.7)	
Age (years)	37.88 ± 14.31	53.39 ± 15.01.^1^	54.82 ± 11.95 ^1^	53.97 ± 11.52 ^1^	49.25 ± 15.17	**<0.001**
Mallampati						**<0.001**
-1	24 (58.5)	6 (18.2)	3 (9.1)	1 (3.2)	34 (24.6)	
-2	9 (22.0)	10 (30.3)	11 (33.3)	5 (16.1)	35 (25.4)	
-3	8 (19.5)	9 (27.3)	10 (30.3)	15 (48.4)	42 (30.4)	
-4	0 (0)	8 (24.2)	9 (27.3)	10 (32.3)	27 (19.6)	
BMI num	24.25 ± 2.98	26.18 ± 2.96 ^1,2^	27.45 ± 2.60 ^1^	28.10 ± 1.99 ^1^	26.34 ± 3.06	**<0.001**
BMI cat n (%)						**<0.001**
BMI 18–25	27 (65.9)	11 (33.3)	7 (21.2)	3 (9.7)	48 (34.8)	
BMI 25–30	14 (34.1)	22 (66.7)	26 (78.8)	28 (90.3)	90 (65.2)	
Neck circumference (cm)	35.78 ± 10.44	35.06 ± 3.29 ^2^	36.91 ± 4.00	39.34 ± 3.24	36.68 ± 6.55	**0.045**
WHR	81.59 ± 9.44	91.30 ± 9.43 ^1,2^	96.27 ± 11.87 ^1^	101.90 ± 8.60 ^1^	91.99 ± 12.45	**<0.001**
Systolic pressure	115.12 ± 9.78	118.94 ± 13.96	121.67 ± 15.39	121.94 ± 12.82	119.13 ± 13.15	0.090
Diastolic pressure	71.34 ± 7.83	76.21 ± 9.27	77.27 ± 11.26 ^1^	77.42 ± 9.74 ^1^	75.29 ± 9.76	**0.018**

Data are expressed as mean ± SD (standard deviation) and as number-n (percentage-%). OSAS severity was classified as none (AHI < 5)-controls, mild (AHI 5–15), moderate (AHI 15–30), and severe (AHI > 30). * Shows the percentage in relation to the total number of the study respondents. ^1^ *p* < 0.05 when the variable in the severe, moderate, or mild OSAS group was compared to the control group. ^2^ *p* < 0.05 when the variable in the mild OSAS group was compared to the severe OSAS group. Significant *p*-values are marked in bold. BMI: Body Mass index; WHR—waist to hip ratio; OSAS—obstructive sleep apnea syndrome.

**Table 2:** Comparison of sleep characteristics between controls and OSAS groups.

Variables	Controls (n = 41)	Mild OSAS (n = 33)	Moderate OSAS (n = 33)	Severe OSAS (n = 31)	*p*-Value
Total sleep time (min)	386.18 ± 70.86	287.43 ± 46.08	387.76 ± 51.49	379.06 ± 51.77	0.921
Sleep efficiency (%)	81.07 ± 13.27	85.13 ± 9.17	84.72 ± 7.37	81.24 ± 9.89	0.209
Latency to sleep	45.14 ± 49.27	26.26 ± 18.83	31.50 ± 31.62	41.23 ± 41.24	0.137
REM (%)	22.64 ± 7.83	22.79 ± 7.73	23.66 ± 6.20	21.10 ± 7.41	0.574
NREM (%)	77.36 ± 7.83	76.76 ± 7.96	76.34 ± 6.21	78.92 ± 7.39	0.530
Slow wave sleep (%) Median (25th–75th perc)	17.1 (8.9–26.9)	12.6 (6.95–16.29) ^1,2^	9.9 (7.15–14.15) ^1^	4.6 (0–12.1) ^1^	**<0.001**
AHI (events/h)	2.7 (1.8–4)	9 (7–11.75) ^1,2^	22 (19.1–25.35) ^1,2^	46.3 (34.2–71.9) ^1^	**<0.001**
AHI REM (events/h)	4.7 (2.7–7.1)	17 (8.6–23.2) ^1,2^	28.7 (19.45–41.20) ^1,2^	54.3 (47.1–66.3) ^1^	**<0.001**
AHI NREM (events/h)	2.1 (0.92–3.4)	6.5 (4.52–8.2) ^2^	19.6 (15.17–25) ^1,2^	49.6 (39–83.6) ^1^	**<0.001**
Arousals index	7.1 (3.55–10.6) ^2,3^	10.4 (6.1–15.55) ^2,3^	22.6 (15.35–26.2) ^2^	37.7 (28.3–70.4) ^3^	**<0.001**
SpO_2_ min. (%)	91.32 ± 2.94 ^2,3^	87.61 ± 3.53 ^2,3^	82.97 ± 5.65 ^2^	76.35 ± 10.83 ^3^	**<0.001**
SpO_2_ ave. (%)	95.50 ± 1.51 ^2,3^	94.19 ± 1.80 ^2^	93.20 ± 2.10 ^2^	89.85 ± 5.09	**<0.001**
ODI (events/h)	2.13 ± 1.70 ^2,3^	7.73 ± 4.84 ^2,3^	19.96 ± 7.97 ^2^	50.74 ± 24.56 ^3^	**<0.001**
ESS	6 (3–9)	10 (6–16) ^1^	9 (6–12) ^1^	9 (7–14) ^1^	**0.008**
Stop Bang	2 (1–4)	4 (3–5) ^1,2^	4 (3–5) ^1,2^	5 (4–6) ^1^	**<0.001**

Data are expressed as mean ± SD (standard deviation) and as median with a range that includes the 25th–75th percentile. OSA severity was classified as none (AHI < 5)-controls, mild (AHI 5–15), moderate (AHI 15–30), and severe (AHI > 30). ^1^ *p* < 0.05 when the variable in the severe, moderate, or mild OSAS group was compared to the control group; ^2^ *p* < 0.05 when the variable in the moderate and mild OSAS group or control group was compared to the severe OSAS group. ^3^ *p* < 0.05 when the variable in the severe and mild OSAS group or control group was compared to the moderate OSAS group. Significant *p*-values are marked in bold. OSAS—obstructive sleep apnea syndrome; AHI—apnea/hypopnea index; REM—rapid eye movement stage of the sleep; NREM—non-rapid eye movement stage of the sleep; SpO_2_ min.—minimum oxyhemoglobin saturation; SpO_2_ ave.—average oxyhemoglobin saturation; ODI—oxygen desaturation index; ESS—Epworth sleepiness scale; Stop Bang questionnaire.

**Table 3:** Comparison of laboratory parameters between controls and OSAS groups.

Variables	Controls (n = 41)	Mild OSAS (n = 33)	Moderate OSAS (n = 33)	Severe OSAS (n = 31)	*p*-Value
pO_2_ (kPa)	11.75 ± 0.89	11.39 ± 1.35	11.17 ± 1.21	10.95 ± 1.18 ^1^	**0.026**
pCO_2_ (kPa)	5.05 ± 0.38	5.06 ± 0.40	4.98 ± 0.36	4.86 ± 0.47	0.136
pH value	7.40 ± 0.03	7.40 ± 0.02	7.41 ± 0.03 ^1^	7.41 ± 0.02	**0.033**
SpO_2_ (%)	96.73 ± 0.63	96.40 ± 1.12	96.15 ± 1.15	96.11 ± 0.99 ^1^	**0.027**
HCO_3_ (mmol/L)	23.35 ± 1.75	23.57 ± 1.55	24.05 ± 1.81	23.29 ± 1.26	0.212
Cholesterol (mmol/L)	4.91 ± 1.05	5.15 ± 0.90	5.08 ± 1.00	5.29 ± 0.93	0.424
HDL-C (mmol/L)	1.55 ± 0.32 ^2^	1.46 ± 0.35 ^2^	1.37 ± 0.39	1.22 ± 0.33	**0.001**
Triglycerides (mmol/L), (median, 25th–75th perc)	1.0 (0.8–1.2)	1.46 (1.06–1.73) ^2^	1.58 (1.06–2.07) ^1^	1.99 (1.24–2.88) ^1^	**<0.001**
LDL-C (mmol/L)	2.89 ± 0.94	3.01 ± 0.79	2.84 ± 1.10	3.01 ± 1.07	0.849
VLDL (mmol/L)	0.03 ± 0.12	0.24 ± 0.36	0.46 ± 0.92 ^1^	0.36 ± 0.47	**0.005**
Glucose (mmol/L)	5.3 (5.1–5.4)	5.5 (5.1–5.95) ^2^	5.8 (5.4–6.2) ^1^	5.7 (5.4–6.5) ^1^	**<0.001**
Insulin (mIU/L)	7.46 ± 3.28	8.01 ± 3.64 ^2^	10.00 ± 5.78	11.12 ± 5.82 ^1^	**0.004**
HOMA index	1.07 ± 0.49	1.19 ± 0.55 ^2^	1.50 ± 0.87	1.67 ± 0.91 ^1^	**0.002**

Data are expressed as mean ± SD (standard deviation) and as median with a range that includes the 25th–75th percentile. OSA severity was classified as none (AHI < 5)-controls, mild (AHI 5–15), moderate (AHI 15–30), and severe (AHI > 30). ^1^ *p* < 0.05 when the variable in the severe or moderate OSAS group was compared to the control group, ^2^ *p* < 0.05 when the variable in the mild OSAS group or control group was compared to the severe OSAS group. Significant *p*-values are marked in bold. OSAS—obstructive sleep apnea syndrome; pO_2_—partial pressure of oxygen; pCO_2_—partial pressure of carbon dioxide; SpO_2_—oxyhemoglobin saturation; HCO_3_—bicarbonate; HDL-C—high-density lipoprotein cholesterol; LDL-C—low-density lipoprotein cholesterol; VLDL—very low-density lipoprotein; HOMA index—Homeostatic Model Assessment of Insulin Resistance.

**Table 4:** Representation of comorbidities in groups of subjects n (%).

Variables	Controls (n = 41)	Mild OSAS (n = 33)	Moderate OSAS (n = 33)	Severe OSAS (n = 31)	Total of the OSAS Patients	Total of the Study Population	*p*-Value
Hypertension	5 (12.2; 3.6) *	19 (57.6; 13.8)	17 (51.5; 12.3)	20 (64.5; 14.5)	56 (40.6)	61 (44.2)	**<0.001**
Heart failure	0 (0.0; 0.0)	0 (0.0; 0.0)	3 (9.1; 2.2)	0 (0.0; 0.0)	3 (2.2)	3 (2.2)	0.036
Coronary disease	0 (0.0; 0.0)	1 (3.0; 0.7)	1 (3.0; 0.7)	1 (3.2; 0.7)	3 (2.2)	3 (2.2)	0.700
History of myocardial infarction	1 (2.4. 0.7)	1 (3.0; 0.7)	1 (3.0. 0.7)	1 (3.2; 0.7)	3 (2.2)	4 (2.9)	1.000
Diabetes mellitus	0 (0.0; 0.0)	1 (3.0; 0.7)	4 (12.1; 2.9)	5 (16.1; 3.6)	10 (7.2)	10 (7.2)	**0.015**
Hyperlipidemia	13 (31.7; 9.4)	20 (60.6; 14.5)	28 (84.8; 20.3)	26 (83.9; 18.8)	74 (53.6)	87 (63.0)	**<0.001**
Hypothyroidism	2 (4.9; 1.4)	2 (6.1; 1.4)	1 (3.0; 0.7)	1 (3.2; 0.7)	4 (2.9)	6 (4.3)	1.000
Asthma	6 (14.6; 4.3)	8 (24.2; 5.8)	2 (6.1; 1.4)	5 (16.1; 3.6)	15 (10.9)	21 (15.2)	0.239
GERD	5 (12.2; 3.6)	8 (24.2; 5.8)	8 (24.2; 5.8)	5 (16.1; 3.6)	21 (15.2)	26 (18.8)	0.478
COPD	2 (4.9; 1.4)	2 (6.1; 1.4)	1 (3.0; 0.7)	1 (3.2; 0.7)	4 (2.9)	6 (4.3)	1.000
Allergic rhinitis	13 (31.7; 9.4)	9 (27.3; 6.5)	5 (15.2; 3.6)	7 (22.6; 5.1)	21 (15.2)	34 (24.6)	0.414
Deviation of the nasal septum	8 (19.5; 5.8)	9 (27.3; 6.5)	7 (21.2; 5.1)	13 (41.9; 9.4)	29 (21.0)	37 (26.8)	0.156
Smoking, n (%)	11 (26.8; 8.0)	9 (27.3; 6.5)	10 (30.3; 7.2)	10 (32.3; 7.2)	29 (21.0)	40 (29.0)	0.390
Snoring							**<0.001**
-mild	39 (95.1; 28.3)	15 (45.5; 10.9)	6 (18.2; 4.3)	4 (12.9; 2.9)	25 (18.1)	64 (46.4)	
-moderate	2 (4.9; 1.4)	12 (36.4; 8.7)	15 (45.5; 10.9)	15 (48.4; 10.9)	42 (30.4)	44 (31.9)	
-loud	0 (0.0; 0.0)	6 (18.2; 4.3)	12 (36.4; 8.7)	12 (38.7; 8.7)	30 (21.7)	30 (21.7)	

* The first percentage in parentheses indicates the ratio within the control group or OSAS groups, and the second percentage in parentheses indicates the ratio to the total number of respondents. OSA severity was classified as none (AHI < 5)-controls, mild (AHI 5–15), moderate (AHI 15–30), and severe (AHI > 30). *p* < 0.05 was statistically significant when the variable in the severe or moderate OSAS group was compared to the control group. Significant *p*-values are marked in bold. GERD—Gastroesophageal reflux disease; COPD—Chronic obstructive pulmonary disease.

**Table 5:** Comparison of SCORE-2 between controls and OSAS groups.

Variable	Controls (n = 36)	Mild OSAS (n = 24)	Moderate OSAS (n = 22)	Severe OSAS (n = 24)	*p*-Value
SCORE-2 CV risk Median (25th–75th perc)	2 (1–4.75)	6.25 (3–13.75) ^1^	7.25 (4.25–16) ^1^	9.75 (5.5–12) ^1^	**<0.001**

Data are expressed as median with a range that includes the 25th–75th percentile. OSA severity was classified as none (AHI < 5)-controls, mild (AHI 5–15), moderate (AHI 15–30), and severe (AHI > 30). ^1^ *p* < 0.05 was statistically significant when the variable in the severe, moderate, or mild OSAS group was compared to the control group. SCORE-2—Systematic Coronary Risk Evaluation, CV—cardiovascular risk.

**Table 6:** Comparison of the Charlson Comorbidity Index between controls and OSAS patients.

Variables	Controls (n = 41)	Total of the OSAS Patients (n = 97)	*p*-Value
Charlson Index n (%)			**0.001**
0–1	35 (85.4; 25.4) *	50 (51.5; 36.2)	
2–3	5 (12.2; 3.6)	38 (39.2; 27.5)	
≥4	1 (2.4; 0.7)	9 (9.3; 6.5)	
CCI 10-year survival Median (25th–75th perc)	98 (96–98)	96 (90–98)	**<0.001**

* The first percentage in parentheses indicates the ratio within the control group or within the OSAS group, and the second percentage in parentheses indicates the ratio to the total number of respondents. The Charlson index was significantly higher in the OSAS group of the patients compared to the controls (*p* = 0.001). CCI 10-year survival score was significantly lower in the OSAS group of the patients compared to the controls (*p* < 0.001). Data are expressed as median with a range that includes the 25th–75th percentile. CCI 10-year survival—Charlson Comorbidity Index 10-year survival.

**Table 7:** Association between SCORE-2 and Charlson Comorbidity Index with other variables.

	SCORE2		Charlson Comorbidity Index	
Variable	r	*p*	r	*p*
Gender	0.208 *	0.033	0.197 *	0.021
Age	0.817 **	0.000	0.751 **	0.000
ESS	0.182	0.061	0.171 *	0.045
Stop Bang	0.470 **	0.000	0.410 **	0.000
Mallampati	0.363 **	0.000	0.257 **	0.002
BMI	0.284 **	0.003	0.133	0.119
Waist circumference	0.280 **	0.004	0.164	0.055
snoring	0.378 **	0.000	0.140	0.102
Slow wave sleep	−0.258 **	0.008	−0.186 *	0.029
AHI	0.232 *	0.017	0.039	0.650
AHI REM	0.328 **	0.001	0.181 *	0.034
AHI NREM	0.207 *	0.033	0.013	0.881
SpO_2_ min	−0.247 *	0.011	−0.069	0.423
SpO_2_ mean	−0.249 *	0.010	−0.064	0.458
ODI	0.235 *	0.015	0.040	0.645
Heart rate min	0.212 *	0.029	0.223 *	0.009
Heart rate max	−0.199 *	0.041	−0.248	0.003
pO_2_	−0.282 **	0.003	−0.361 *	0.000
pCO_2_	−0.242 *	0.013	−0.076	0.376
pH value	0.316 **	0.001	0.279 **	0.001
SpO_2_	−0.212 *	0.029	−0.342**	0.000

ESS-Epworth sleepiness scale; BMI—Body Mass Index; AHI—apnea/hypopnea index; AHI REM—apnea/hypopnea index in the rapid eye movement stage of the sleep; AHI NREM—apnea/hypopnea index in the non-rapid eye movement stage of the sleep; SpO_2_ min—minimum oxyhemoglobin saturation; SpO_2_ mean—average oxyhemoglobin saturation; ODI—oxygen desaturation index; pO_2_—partial pressure of oxygen; pCO_2_—partial pressure of carbon dioxide; pH value—potential of hydrogen. Significant correlations are marked as * *p* < 0.05 and ** *p* < 0.01.

**Table 8:** Multivariate linear regression model for prediction OSAS severity.

	Mild OSAS			Moderate OSAS			Severe OSAS		
**Variables**	B	SE	*p*	B	SE	*p*	B (SE)	SE	*p*
**Gender**	−1.639	1.150	0.154	**−3.136**	**1.296**	**0.016**	**−3.796**	**1.412**	**0.007**
**Age**	0.076	0.048	0.115	**0.105**	**0.054**	**0.048**	0.104	0.058	0.071
Epworth	0.102	0.079	0.198	0.056	0.091	0.543	0.043	0.100	0.667
Stop Bang	−0.106	0.387	0.784	−0.165	0.433	0.703	0.614	0.481	0.202
**Mallampati**	**0.944**	**0.474**	**0.046**	0.969	0.520	0.062	**1.667**	**0.556**	**0.003**
BMI	−0.046	0.143	0.746	0.130	0.177	0.462	0.094	0.195	0.629
Hypertension	0.993	0.983	0.312	0.020	1.162	0.986	−0.266	1.212	0.826
**Snoring**	**3.221**	**1.071**	**0.003**	**3.992**	**1.111**	**0.000**	**4.112**	**1.132**	**0.000**
pO_2_	0.474	0.416	0.254	0.491	0.446	0.272	0.627	0.477	0.188
pH	−3.589	18.272	0.844	31.693	20.402	0.120	16.445	21.298	0.440
HDL-C	−1.019	1.544	0.509	−0.797	1.723	0.644	−2.450	1.891	0.195
Triglycerides	0.803	1.072	0.454	1.161	1.088	0.286	0.583	1.102	0.596
HOMA index	−0.106	0.761	0.889	0.309	0.839	0.713	0.696	0.857	0.417
SCORE2	−0.085	0.093	0.358	0.068	0.098	0.487	−0.039	0.103	0.704

The multivariate linear regression model was used for the prediction of obstructive sleep apnea severity. In the complete model for the prediction of severe OSAS, three parameters proved to be significant: gender, Mallampati score, and snoring. Significant parameters are marked in bold. BMI—Body Mass Index; pO_2_—partial pressure of oxygen; pH value—potential of hydrogen; HDL-C—high-density lipoprotein cholesterol; HOMA index—Homeostatic Model Assessment of Insulin Resistance; SCORE-2—Systematic Coronary Risk Evaluation

## Data Availability

The data presented in this study are available on request from the corresponding author. The data are not publicly available due to terms of privacy.

## References

[1] Kopitović I. (2011). Respiratorni Poremećaji Tokom Spavanja [Sleep Breathing Disorders].

[2] Jovančević D.M., Kopitović I., Miličić D., Pavlović-Popović Z. (2013). Dijagnostika respiratornih poremećaja tokom spavanja [Diagnostics of Sleep Breathing Disorders]. Pneumon.

[3] Drager L.F., Bortolotto L.A., Figueiredo A.C., Silva B.C., Krieger E.M., Lorenzi-Filho G. (2007). Obstructive sleep apnea, hypertension, and their interaction on arterial stiffness and heart remodelling. Chest.

[4] Lavie L. (2009). Oxidative Stress—A Unifying Paradigm in Obstructive Sleep Apnea and Comorbidities. Prog. Cardiovasc. Dis..

[5] Suzuki Y.J., Jain V., Park A.M., Day R.M. (2006). Oxidative Stress and Oxidant Signaling in Obstructive Sleep Apnea and Associated Cardiovascular Diseases. Free Radic. Biol. Med..

[6] Lam J.C., Yan C.S., Lai A.Y., Tam S., Fong D.Y., Lam B., Ip M.S. (2009). Determinants of Daytime Blood Pressure in Relation to Obstructive Sleep Apnea in Men. Lung.

[7] Punjabi N.M. (2008). The Epidemiology of Adult Obstructive Sleep Apnea. Proc. Am. Thorac. Soc..

[8] Tufik S., Santos-Silva R., Taddei J.A., Bittencourt L.R.A. (2010). Obstructive sleep apnea syndrome in the Sao Paulo Epidemiologic Sleep Study. Sleep Med..

[9] McNicholas W.T. (2019). Obstructive Sleep Apnoea and Comorbidity—An Overview of the Association and Impact of Continuous Positive Airway Pressure Therapy. Expert Rev. Respir. Med..

[10] Punjabi N.M., Caffo B.S., Goodwin J.L., Gottlieb D.J., Newman A.B., O’Connor G.T., Rapoport D.M., Redline S., Resnick H.E., Robbins J.A. (2009). Sleep-disordered breathing and mortality: A prospective cohort study. PLoS Med..

[11] Javaheri S., Barbe F., Campos-Rodriguez F., Dempsey J.A., Khayat R., Javaheri S., Malhotra A., Martinez-Garcia M.A., Mehra R., Pack A.I. (2017). Sleep Apnea: Types, Mechanisms, and Clinical Cardiovascular Consequences. J. Am. Coll. Cardiol.

[12] Kopitovic I., Trajanovic N., Prodic S., Drvenica M.J., Ilic M., Kuruc V., Kojicic M. (2011). The Serbian version of the Epworth Sleepiness Scale. Sleep Breath.

[13] Örnek T., Koçak E., Koçak G., Bakırtaş H., Atmaca H., Can M., Bayraktaroğlu T., Altın R. (2011). Insulin Resistance and Serum Leptin Levels in Men with Obstructive Sleep Apnea Syndrome. Electron J. Gen. Med..

[14] Cizza G., Piaggi P., Lucassen E.A., de Jonge L., Walter M., Mattingly M.S., Kalish H., Csako G., Rother K.I. (2013). Obstructive Sleep Apnea Is a Predictor of Abnormal Glucose Metabolism in Chronically Sleep Deprived Obese Adults. PLoS ONE.

[15] Lee R., McNicholas W.T. (2011). Obstructive Sleep Apnea in Chronic Obstructive Pulmonary Disease Patients. Curr. Opin. Pulm. Med..

[16] McNicholas W.T., Verbraecken J., Marin J.M. (2013). Sleep Disorders in COPD: The Forgotten Dimension. Eur. Respir. Rev..

[17] Durgan D.J., Bryan R.M. (2012). Cerebrovascular Consequences of Obstructive Sleep Apnea. J. Am. Heart. Assoc..

[18] Chen Y.H., Keller J.K., Kang J.H., Hsieh H.J., Lin H.C. (2013). Obstructive sleep apnea and the subsequent risk of depressive disorder: A population-based follow-up study. J. Clin. Sleep Med..

[19] Kendzerska T., Gershon A.S., Hawker G., Leung R.S., Tomlinson G. (2014). Obstructive Sleep Apnea and Risk of Cardiovascular Events and All-Cause Mortality: A Decade-Long Historical Cohort Study. PLoS Med..

[20] Jennum P., Kjellberg J. (2011). Health, Social and Economical Consequences of Sleep-Disordered Breathing: A Controlled National Study. Thorax.

[21] Hopps E., Canino B., Calandrino V., Montana M., Lo Presti R., Caimi G. (2014). Lipid peroxidation and protein oxidation are related to the severity of OSAS. Eur. Rev. Med. Pharmacol. Sci..

[22] Charlson M.E., Pompei P., Ales K.L., MacKenzie C. (1987). Ronald. A New Method of Classifying Prognostic Comorbidity in Longitudinal Studies: Development and Validation. J. Chronic Dis..

[23] Charlson M., Szatrowski T.P., Peterson J., Gold J. (1994). Validation of a Combined Comorbidity Index. J. Clin. Epidemiol..

[24] SCORE2 working group and ESC Cardiovascular risk collaboration (2021). SCORE2 risk prediction algorithms: New models to estimate 10-year risk of cardiovascular disease in Europe. Eur. Heart. J..

[25] Iber C., Ancoli-Israel S., Chesson A.L., Quan S.F., Darien I.L. (2023). The AASM Manual for the Scoring of Sleep and Associated Events: Rules, Therminology and Technical Specification, Version 3.

[26] Hirshkowitz M., Kryger M., Kryger M.H., Roth T., Dement W.C. (2022). Monitoring techniques for Evaluating Suspected Sleep-Related Breathing and cardiovascular Disorders. Principles and Practice of Sleep Medicine.

[27] Matthews D.R., Hosker J.P., Rudenski A.S., Naylor B.A., Treacher D.F., Turner R.C. (1985). Homeostasis Model Assessment: Insulin Resistance and beta-cell Function from Fasting Plasma Glucose and Insulin Concentrations in Man. Diabetologia.

[28] Wolk R., Shamsuzzaman A.S.M., Somers V.K. (2003). Obesity, Sleep Apnea, and Hypertension. Hypertension.

[29] McNicholas W.T., Bonsigore M.R., Management Committee of EU COST ACTION B26 (2007). Sleep Apnoea as an Independent Risk Factor for Cardiovascular Disease: Current Evidence, Basic Mechanisms and Research Priorities. Eur. Respir. J..

[30] Azarbarzin A., Sands S.A., Stone K.L., Taranto-Montemurro L., Messineo L., Terrill P.I., Ancoli-Israel S., Ensrud K., Purcell S., White D.P. (2018). The Hypoxic Burden of Sleep Apnoea Predicts Cardiovascular Disease-Related Mortality: The Osteoporotic Fractures in Men Study and the Sleep Heart Health Study. Eur. Heart. J..

[31] Somers V.K., White D.P., Amin R., Abraham W.T., Costa F., Culebras A., Daniels S., Floras J.S., Hunt C.E., Olson L.J. (2008). American Heart Association Council for High Blood Pressure Research Professional Education Committee, Council on Clinical Cardiology; American Heart Association Stroke Council; American Heart Association Council on Cardiovascular Nursing; American College of Cardiology Foundation. Sleep apnea and cardiovascular disease: An American Heart Association/american College Of Cardiology Foundation Scientific Statement from the American Heart Association Council for High Blood Pressure Research Professional Education Committee, Council on Clinical Cardiology, Stroke Council, and Council On Cardiovascular Nursing. In collaboration with the National Heart, Lung, and Blood Institute National Center on Sleep Disorders Research (National Institutes of Health). Circulation.

[32] Bradley T.D., Floras J.S. (2009). Obstructive Sleep Apnoea and Its Cardiovascular Consequences. Lancet.

[33] Lévy P., Kohler M., McNicholas W.T., Barbé F., McEvoy R.D., Somers V.K., Lavie L., Pépin J.L. (2015). Obstructive sleep apnoea syndrome. Nat. Rev. Dis. Primers.

[34] Jelic S., Padeletti M., Kawut S.M., Higgins C., Canfield S.M., Onat D., Colombo P.C., Basner R.C., Factor P., LeJemtel T.H. (2008). Inflammation, Oxidative Stress, and Repair Capacity of the Vascular Endothelium in Obstructive Sleep Apnea. Circulation.

[35] Lavie L. (2015). Oxidative stress in obstructive sleep apnea and intermittent hypoxia-revisited-the bad ugly and good: Implications to the heart and brain. Sleep Med. Rev..

[36] Senaratna C.V., Perret J.L., Lodge C.J., Lowe A.J., Campbell B.E., Matheson M.C., Hamilton G.S., Dharmage S.C. (2017). Prevalence of Obstructive Sleep Apnea in the General Population: A Systematic Review. Sleep Med. Rev..

[37] Floras J.S. (2009). Hypertension, sleep apnea, and atherosclerosis. Hypertension.

[38] Oldenburg O., Wellmann B., Buchholz A., Bitter T., Fox H., Thiem U., Horstkotte D., Wegscheider K. (2016). Nocturnal Hypoxaemia Is Associated with Increased Mortality in Stable Heart Failure Patients. Eur. Heart. J..

[39] Smagula S.F., Stone K.L., Redline S., Ancoli-Israel S., Barrett-Connor E., Lane N.E., Orwoll E.S., Cauley J.A. (2016). Actigraphy- and Polysomnography-Measured Sleep Disturbances, Inflammation, and Mortality among Older Men. Psychosom Med..

[40] Shivashankar R., Kondal D., Ali M.K., Gupta R., Pradeepa R., Mohan V., Kadir M.M., Narayan K.M.V., Tandon N., Prabhakaran D. (2017). Associations of Sleep Duration and Disturbances with Hypertension in Metropolitan Cities of Delhi, Chennai, and Karachi in South Asia: Cross-Sectional Analysis of the CARRS Study. Sleep.

[41] Kayabekir M., Yağanoğlu M. (2022). The relationship between snoring sounds and EEG signals on polysomnography. Sleep Breath..

[42] Di Mauro P., Cocuzza S., Maniaci A., Ferlito S., Rasà D., Anzivino R., Vicini C., Iannella G., La Mantia I. (2021). The Effect of Adenotonsillectomy on Children’s Behavior and Cognitive Performance with Obstructive Sleep Apnea Syndrome: State of the Art. Children.

[43] Ren R., Covassin N., Zhang Y., Lei F., Yang L., Zhou J., Tan L., Li T., Li Y., Shi J. (2020). Interaction between Slow Wave Sleep and Obstructive Sleep Apnea in Prevalent Hypertension. Hypertension.

[44] Zhang J., Zhuang Y., Wan N., Tang X., Zhou W., Si L., Wang Y., Chen B., Cao J. (2020). Slow-Wave Sleep Is Associated with Incident Hypertension in Patients with Obstructive Sleep Apnea: A Cross-Sectional Study. J. Int. Med. Res..

[45] Hamilton G.S., Meredith I.T., Walker A.M., Solin P. (2009). Obstructive Sleep Apnea Leads to Transient Uncoupling of Coronary Blood Flow and Myocardial Work in Humans. Sleep.

[46] Hamilton G.S., Solin P., Walker A. (2008). Coronary Blood Flow Becomes Uncoupled from Myocardial Work during Obstructive Sleep Apnea in the Presence of Endothelial Dysfunction. Sleep.

[47] Gać P., Urbanik D., Macek P., Martynowicz H., Mazur G., Poręba R. (2022). Coexistence of cardiovascular risk factors and obstructive sleep apnoea in polysomnography. Respir. Physiol. Neurobiol..

[48] Dudenbostel T., Calhoun D.A. (2012). Resistant Hypertension, Obstructive Sleep Apnoea and Aldosterone. J. Hum. Hypertens..

[49] Ahmad M., Makati D., Akbar S. (2017). Review of and Updates on Hypertension in Obstructive Sleep Apnea. Int. J. Hypertens..

[50] Jin Z.N., Wei Y.X. (2016). Meta-analysis of effects of obstructive sleep apnea on the renin-angiotensin-aldosterone system. J. Geriatr. Cardiol..

[51] Stavaras C., Pastaka C., Papala M., Gravas S., Tzortzis V., Melekos M., Seitanidis G., Gourgoulianis K.I. (2012). Sexual function in pre- and post-menopausal women with obstructive sleep apnea syndrome. Int. J. Impot. Res..

[52] Milicic Stanic B., Ilincic B., Zeravica R., Milicic Ivanovski D., Cabarkapa V., Mijovic R. (2022). The Importance of Correlation between Aldosterone and Parathyroid Hormone in Patients with Primary Hyperparathyroidism. Int. J. Endocrinol..

[53] Basoglu O.K., Tasbakan M.S., Kayikcioglu M. (2023). Dyslipidemia prevalence in non-obese non-diabetic patients with obstructive sleep apnea: Does sex matter?. J. Clin. Sleep Med..

[54] Muraki I., Wada H., Tanigawa T. (2018). Sleep Apnea and Type 2 Diabetes. J. Diabetes Investig..

[55] Kendzerska T., Gershon A.S., Hawker G., Tomlinson G., Leung R.S. (2014). Obstructive Sleep Apnea and Incident Diabetes. A Historical Cohort Study. Am. J. Respir. Crit. Care Med..

[56] Appleton S.L., Vakulin A., McEvoy R.D., Wittert G.A., Martin S.A., Grant J.F., Taylor A.W., Antic N.A., Catcheside P.G., Adams R.J. (2015). Nocturnal Hypoxemia and Severe Obstructive Sleep Apnea are Associated with Incident Type 2 Diabetes in a Population Cohort of Men. J. Clin. Sleep Med..

[57] Yang W., Cai X., Hu J., Wen W., Mulalibieke H., Yao X., Yao L., Zhu Q., Hong J., Luo Q. (2023). The Metabolic Score for Insulin Resistance (METS-IR) Predicts Cardiovascular Disease and Its Subtypes in Patients with Hypertension and Obstructive Sleep Apnea. Clin. Epidemiol.

[58] Ormazabal V., Nair S., Elfeky O., Aguayo C., Salomon C., Zuñiga F.A. (2018). Association between Insulin Resistance and the Development of Cardiovascular Disease. Cardiovasc. Diabetol..

[59] Savage D.B., Petersen K.F., Shulman G.I. (2007). Disordered Lipid Metabolism and the Pathogenesis of Insulin Resistance. Physiol. Rev..

[60] Marin J.M., Carrizo S.J., Vicente E., Agusti A.G. (2005). Long-Term Cardiovascular Outcomes in Men with Obstructive Sleep Apnoea-Hypopnoea with or without Treatment with Continuous Positive Airway Pressure: An Observational Study. Lancet.

[61] Yan X., Wang L., Liang C., Zhang H., Zhao Y., Zhang H., Yu H., Di J. (2022). Development and assessment of a risk prediction model for moderate-to-severe obstructive sleep apnea. Front. Neurosci..

[62] Ye P., Qin H., Zhan X., Wang Z., Liu C., Song B., Kong Y., Jia X., Qi Y., Ji J. (2023). Diagnosis of Obstructive Sleep Apnea in Children Based on the XGBoost Algorithm Using Nocturnal Heart Rate and Blood Oxygen Feature. Am. J. Otolaryngol..

[63] Maniaci A., Riela P.M., Iannella G., Lechien J.R., La Mantia I., De Vincentiis M., Cammaroto G., Calvo-Henriquez C., Di Luca M., Chiesa Estomba C. (2023). Machine Learning Identification of Obstructive Sleep Apnea Severity through the Patient Clinical Features: A Retrospective Study. Life.

[64] Xie C., Zhu R., Tian Y., Wang K. (2017). Association of obstructive sleep apnoea with the risk of vascular outcomes and all-cause mortality: A meta-analysis. BMJ Open.

[65] Jonas D.E., Amick H.R., Feltner C., Weber R.P., Arvanitis M., Stine A., Lux L., Middleton J.C., Voisin C., Harris R.P. (2017). Screening for Obstructive Sleep Apnea in Adults: An Evidence Review for the U.S. Preventive Services Task Force.

[66] Ruel G., Martin S.A., Lévesque J.-F., Wittert G.A., Adams R.J., Appleton S.L., Shi Z., Taylor A.W. (2018). Association between Multimorbidity and Undiagnosed Obstructive Sleep Apnea Severity and Their Impact on Quality of Life in Men over 40 Years Old. Glob. Health Epidemiol. Genom..

[67] Chiang C.L., Chen Y.T., Wang K.L., Su V.Y., Wu L.A., Perng D.W., Chang S.C., Chen Y.M., Chen T.J., Chou K.T. (2017). Comorbidities and risk of mortality in patients with sleep apnea. Ann. Med..

[68] Archontogeorgis K., Voulgaris A., Nena E., Strempela M., Karailidou P., Tzouvelekis A., Mouemin T., Xanthoudaki M., Steiropoulos S., Froudarakis M.E. (2018). Cardiovascular Risk Assessment in a Cohort of Newly Diagnosed Patients with Obstructive Sleep Apnea Syndrome. Cardiol. Res. Pract..

[69] Chuang H.-H., Huang C.-G., Chuang L.-P., Huang Y.-S., Chen N.-H., Li H.-Y., Fang T.-J., Hsu J.-F., Lai H.-C., Chen J.-Y. (2020). Relationships Among and Predictive Values of Obesity, Inflammation Markers, and Disease Severity in Pediatric Patients with Obstructive Sleep Apnea Before and After Adenotonsillectomy. J. Clin. Med..

